# LC-MS/MS analysis and evaluation of the anti-inflammatory activity of components from BushenHuoxue decoction

**DOI:** 10.1080/13880209.2017.1285327

**Published:** 2017-02-05

**Authors:** Dongdong Sun, Qiuying Yan, Xiaofang Xu, Weixing Shen, Changliang Xu, Jiani Tan, Haibin Zhang, Liu Li, Haibo Cheng

**Affiliations:** a Translational Medicine Center of Nanjing University of Chinese Medicine, Nanjing, China;; b Jiangsu Collaborative Innovation Center of Traditional Chinese Medicine (TCM) Prevention and Treatment of Tumor, Nanjing, China;; c Key Laboratory of Famous Doctors' Proved Recipe Evaluation and Transformation of State Administration of Traditional Chinese Medicine, Nanjing, China

**Keywords:** ELISA, osteoarthritis, qualitative analysis, simultaneously determine

## Abstract

**Context:** BushenHuoxue decoction (BSHXD) is a Chinese medicine prescription, which is composed of nine Chinese medical materials, used to treat osteoarthritis (OA).

**Objective:** This study develops sensitive and convenient LC-MS/MS methods to analyze chemical components from BSHXD, and assess the anti-inflammatory activities thereof.

**Materials and methods:** The chemical composition from BSHXD water extract was qualitative analyzed by high-performance liquid chromatography coupled with electrospray ionization quadrupole time-of-flight mass spectrometry (HPLC-ESI-Q-TOF-MS). Twelve reference compounds were analyzed by UPLC-ESI-MS/MS. Anti-inflammatory activities of target components were assessed by ELISA at 20 and 100 μg/mL.

**Results:** It is the first time that 88 compounds were qualitatively identified from BSHXD, of which 12 with potential in treating OA according to the literature were quantified. Within BSHXD the contents of quercetin, isopsoralen, icarisideII, osthole, and isoimperatorin increased remarkably compared with those in single herb which make up BSHXD, the contents were 0.1999, 0.4634, 0.0928, 0.5364, and 0.1487 mg/g. ELISA data displayed that BSHXD and the five compounds mentioned inhibited the expressions of TNF-α, IL-6 and NO released from LPS-stimulated RAW264.7 cell, with maximum inhibition rates of 104.05% (osthole, 100 μg/mL), 100.03% (osthole, 100 μg/mL), and 93.46% (isopsoralen, 20 μg/mL), respectively.

**Discussion and conclusion:** Content changes of 12 compounds in BSHXD and single herbs which comprise the prescription were measured and analyzed. Contents of five compounds increased may be explained by solubilization between drugs and chemical reaction. ELISA results reported that the increased contents of the five compounds could inhibit expression of the inflammatory factors.

## Introduction

BushenHuoxue Decoction (BSHXD), which is applied to treat osteoarthritis (OA), originates from a commonly used recipe for Jiangsu Province Hospital of Traditional Chinese Medicine. BSHXD was composed of *Angelicae Pubescentis Radix, Taxilli Herba, Achyranthis Bidentatae Radix, Epimedii Folium, Angelicae Sinensis Radix, Chuanxiong Rhizoma, Paeoniae Radix Alba, Polygoni Cuspidati Rhizoma Et Radix,* and *Arisaematis Rhizoma Preparatum.*

OA which is also called hypertrophic arthritis or degenerative arthritis was frequently occurred and refractory. It is expressed by arthralgia, stiffness, and deformity (Zhang et al. 2007; Juhl et al. 2014). Depending on the basic theories of Chinese medicine, it is a type of bone obstruction disease. The principal pathological symptom of OA is lesion of cartilage tissue. Cytokine plays an important role in the pathogenesis of OA by promoting catabolism of cartilage matrix (Schable 2014; Yang et al. 2014; Cornejo et al. 2015).

High-performance liquid chromatography coupled with electrospray ionization quadrupole time-of-flight mass spectrometry (HPLC-ESI-Q-TOF-MS) was established for qualitative analysis on chemical compounds of BSHXD. Eighty-eight compounds were confirmed by comparing its retention time and MS spectrum with the corresponding reference compound. Through the review of literature, 12 active compounds with potential in treating OA and inhibiting chondrocyte apoptosis were found from these 88 compounds. UPLC-ESI-TQ-MS method was also established to simultaneously determine and compare the contents of these 12 compounds in BSHXD and in single herb decoction. The results showed that the contents of five compounds, which were quercetin, isopsoralen, icariside II, osthole, and isoimperatorin, increased remarkably in BSHXD. To evaluate the anti-inflammatory activity and explore the therapeutic mechanism of BSHXD against OA, the effect of above five compounds on TNF-α, IL-6, and NO released by macrophage was assayed.

## Materials and methods

### Chemicals, reagents, and samples

The reference compounds of catechin (no. 10144-201209), paeoniflorin (no. 0736-9811), hyperoside (no. 10228-201204), ferulic acid (no. 0773-9910), polydatin (no. 10201-201209), quercetin (no. 081-9003), resveratrol (no. 10040-201201), psoralen (no. 739-8701), isopsoralen (no. 0739-200108), icariside II (no. 20264-201201), osthole (no. 0822-9802), and isoimperatorin (no. 10531-201203) were obtained from the National Institutes for the Control of Pharmaceutical and Biological Products (Beijing, China). LPS (1 mg/mL, Sigma-Aldrich, St. Louis, MO), 1640 medium (Gibico, Waltham, MA), FBS (Sijiqing Co., Ltd, Shanghai, China), Pancreatin (no. 27250018, Gibico, Waltham, MA), DMSO (no. 20110105, Lingfeng Co., Ltd, Shanghai, China), TNF-α ELISA kit (no. EM004-96, 96t, Kesai Biological Products Co., Shanghai, China), IL-6 ELISA kit (no. EM008-96, 96t, Kesai Biological Products Co., Shanghai, China), and NO kit (no. S0021-2, 200t, Beyotime Institute of Biotechnology, Jiangsu, China).

Methanol and acetonitrile were of HPLC grade and purchased from Hanbang Technology Co., Ltd. (Jiangsu, China). Ultra-pure water was obtained by the EPED super-purification system (Nanjing EPED Co., Ltd, Nanjing, China). All other chemicals and solvents used in this study were of analytical grade. The herbal materials were purchased in June 2011 from Bozhou Medicinal Material Company (Bozhou, China) and authenticated by Prof. Jianwei Chen of Nanjing University of Chinese Medicine, Nanjing, China. Voucher specimens were deposited at Key Laboratory of Famous Doctors' Proved Recipe Evaluation and Transformation of State Administration of Traditional Chinese Medicine.

## Instrument and LC-MS/MS conditions

### Instruments

RE-52A rotary evaporator (Shanghai Yarong Biochemistry Instrument Factory, Shanghai, China), hypothermia centrifugal machine (model TGL16, Changsha Xiangzhi, Changsha, China), water bath (model SY-1220, Crystal, Santa Clara, CA), super clean bench (model 1300 SERIES A2, Thermo Scientific, Waltham, MA), CO_2_ incubator (Thermo Scientific SERIES II WATER JACKET), and enzyme-labelling instrument (Thermo Scientific, Waltham, MA).

### Chromatographic conditions

HPLC analysis was performed on a Shimadzu LC-20A HPLC system (Shimadzu, Kyoto, Japan) equipped with a binary pump, an online degasser, an autosampler, and a column oven, using a Hanbon Lichrospher^TM^ HPLC C18 column (4.6 × 250 mm, 5 μm). The mobile phase was methanol (A) and 0.1% aqueous formic acid (v/v, B), with a gradient elution of 10% A in 0–5 min, 10–30% A in 5–10 min, 30–85% A in 10–15 min, 85–100% A in 15–20 min, 100% A in 20–23 min, 100–10% A in 23–30 min, at a flow rate of 1.0 mL/min, the injection volume was 10 μL, and the temperature of the column was maintained at 40 °C.

UPLC analysis was performed on a Waters ACQUITY UPLC system (Waters, Milford, MA) equipped with a binary pump, an online degasser, an autosampler, and a column oven, using a BEH C18 column (2.1 × 100 mm I.D., 1.7 μm, Waters, Milford, MA). The mobile phase consisted of 0.1% aqueous formic acid (v/v, A) and acetonitrile (B), with a gradient program of 90–65% A in 0–7 min, 65–40% A in 7–11 min, 40–0% A in 11–14 min, 0–0% A in 14–17 min, 0–90% A in 17–18 min, at a flow rate was 0.4 mL/min, the injection volume was 2 μL, and the temperature of the column was maintained at 35 °C.

### Mass-spectrometry conditions

The qualitative analysis was performed on an AB SCIEX Triple Tof 5600 (AB SCIEX, Foster City, CA) equipped with an electrospray ionization (ESI) source, and the ESI source was set in positive and negative mode. The scanning mode was set in multiple reaction monitoring (MRM) mode. The ion spray (IS) voltage was set at −4500.00 V; declustering potential (DP), −80 V; collision (CE), −30.0 V; ion source gas 1, 55.00 psi; ion source gas 2, 55.00 psi; CUR, 40.00 psi; TEM, 500.00 °C; TOF MASSES (DA), Min =100.0000, Max =1200.0000; collisional excitation scanning (CES), 20.0.

The quantitative analysis was performed on a Waters-Xevo TQ, tri-stage quadrupole mass spectrometer system (Waters, Milford, MA) equipped with an ESI source, and the ESI source was set in positive and negative mode. The scanning mode was established in MRM mode. The capillary voltage was 3 kV, ion source temperature was 150 °C, the dry gas flow was 1000 L/h, dry heater was 550 °C, the cone gas flow was 50 L/h, and the collision gas flow was 0.15 mL/min.

### Preparation of sample solutions for LC-MS/MS analysis

*Angelicae Pubescentis Radix* 400 g, *Taxilli Herba* 400 g*, Achyranthis Bidentatae Radix* 600 g*, Epimedii Folium* 600 g*, Angelicae Sinensis Radix* 600 g*, Chuanxiong Rhizoma* 600 g*, Paeoniae Radix Alba* 600 g*, Polygoni Cuspidati Rhizoma Et Radix* 600 g, and *Arisaematis Rhizoma Preparatum* 600 g were mixed together. The mixture was decocted with 30 L water three times (1, 1, and 0.5 h). The filtrates from each decoction were consolidated and concentrated to 1000 mL, and added 1100 mL 95% ethanol, suspension was centrifuged at 5000 rpm for 10 min after 48 h standing. The supernatant was dried on a water bath, and dissolved 0.0001 g dried supernatant in 50% methanol to 100 mL, then filtered through 0.22 μm membrane filter to produce stock solutions. The reference compounds were accurately weighed and dissolved in 50% methanol to produce stock reference solutions. The above stock solutions was stored at 4 °C and brought to room temperature before use.

## Enzyme-linked immunosorbent assay

### Cell culture

The murine macrophages RAW264.7 were cultured in 1640 culture medium with 10% FBS in an incubator containing 5% CO_2_ at 37 °C. The cells were digested with trypsin when they grew to an appropriate amount, and placed into the wells of a 48-well plate at 200 μL/well (1 × 10^5^ cells/well) and cultured for 24 h in the sterilized incubator.

### Grouping and administration

LPS was diluted with 1640 culture medium to 500 ng/mL, BSHXD and compound samples were dissolved in DMSO at 1 × 10^5^ μg/mL and diluted to 100 and 20 μg/mL with the culture medium. Above samples were added to each of control group, LPS group, and sample groups (LPS + sample 20 μg/mL, LPS + sample 100 μg/mL) in an amount of 300 μL. The 48-well plate was incubated another 24 h in the sterilized incubator.

### Sample detection

The supernatant was collected after centrifuging at 10,000 rpm for 2 min at 4 °C. The supernatant was treated and the absorbance was measured at 450 nm (TNF-α, IL-6) and 540 nm (NO) using an enzyme-labelling instrument according to the instruction of the manufacturer, respectively. Cytokine concentrations were calculated by a calibration curve prepared by standard concentrations as *X*-axis, and OD values as *Y*-axis.

### Data analysis

Inhibitory rate (%) = 100%− (*C*_LPS + sample_−*C*_LPS_)/(*C*_LPS_−*C*_untreated_), *C* is the cytokine concentration. All samples were assayed in triplicate. The results were presented as means ± standard deviations (SD). Statistical analyses were performed using a one-way analysis of variance ANOVA test (SPSS v.16.0, SPSS Inc., Chicago, IL), followed by Student’s two-tailed unpaired *t-*test. *p* < 0.05 was considered as the statistically significant.

## Results and discussion

### Optimization of ion condition

In order to determine appropriate ion condition for Q-TOF and TQ mass spectrometer, all the analytes were detected under affuse mode, and the fragment ions were automatically collected under MRM scanning mode, with optimal cone voltage and collision energies.

### Selection of mobile phases

A series of experiments were carried out with different mobile phases, for example methanol/water, methanol/acetonitrile–water, methanol/acetonitrile–0.1% formic acid–water, methanol/acetonitrile–0.05% aqueous formic acid. It turned out that the best chromatographic peak was obtained when using methanol–0.1% aqueous formic acid solution and 0.1% aqueous formic acid solution–acetonitrile as the mobile phases for HPLC-ESI-Q-TOF-MS and UPLC-ESI-TQ-MS, respectively.

### Qualitative analysis

**Figure 1.**
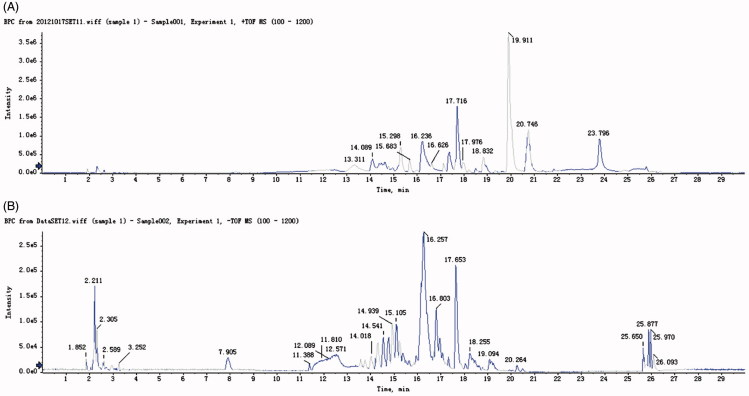
Total ion flow chart of positive ion (A) and negative ion (B) mode in BSHXD.

Qualitative analysis on compounds in BSHXD was achieved by HPLC-ESI-Q-TOF-MS, the total ion flow chart of positive and negative ion modes is shown in Figure 1. These peaks showed different molecular ion the MS^2^ spectra, which exhibited a fragmentation pathway. According to measured molecular weight, theoretical molecular weight, fragment ion, elemental analysis, and compared with relevant literature data, 88 compounds from BSHXD were detected and confirmed. The mass error for molecular ions was in ±5 ppm. The detailed spectral data are presented in Table 1. The results provided some evidence of the material basis for the BSHXD.

**Table 1.** Qualitative analysis on chemical compounds in BSHXD.

		Negative ion (*m/z*)			Positive ion (*m/z*)					
No.	TR/min	Measured molecular weight	Theoretical molecular weight	ppm	Measured molecular weight	Theoretical molecular weight	ppm	Fragment ion	Molecular formula	Compound
1	1.82				175.1186	175.119	−0.23	(+)130,116,112	C_6_H_14_N_4_O_2_	Arginine
2	2.18	341.1091	341.1089	0.06	365.1053	365.1054	−0.03	(−)221,179,161,119,113,(+)203	C_12_H_22_O_11_	Sucrose
3	2.29	179.0571	179.0561	0.59				(−)131,101	C_6_H_12_O_6_	Glucose
4	2.3	179.0576	179.0561	0.84				(−)161,134	C_6_H_12_O_6_	Inositol
5	2.34				118.0863	118.0863	0	(+) 118	C_5_H_11_NO_2_	Valine
6	2.38				138.0548	138.055	−0.14	(+) 120	C_7_H_7_NO_2_	Trigonelline
7	2.38				176.1025	176.103	−0.28	(+) 130,	C_6_H_13_N_3_O_3_	Citrulline
8	2.49				144.1007	144.1019	−0.83	(+) 102	C_7_H_13_NO_2_	Stachydrine
9	2.81				156.0761	156.0768	−0.45	(+) 110	C_6_H_9_N_3_O_2_	L-Histidine
10	2.86	243.0632	243.0636	−0.16				(−)130,110	C_9_H_12_N_2_O_6_	Uridine
11	3.7	169.0152	169.0143	0.53				(−)125,107	C_7_H_6_O_5_	Gallic acid
12	6.52	153.0213	153.0193	1.3				(−)108	C_7_H_6_O_4_	2,4-Dihydroxybenzoic acid
13	7.75	543.1173	543.1178	−0.09				(−)421,259,121	C_23_H_28_O_13_S	Paeoniflorin sulphurous acid ester
14	8.52				127.04	127.039	0.79	(+)109	C_6_H_6_O_3_	5-Hydroxymethyfurfural
15	8.65				268.1032	268.1027	0.18	(+)136,119	C_10_H_13_N_5_O_4_	Adenosine
16	9.04	495.15	495.1508	−0.16	519.1501	519.1473	0.54	(−)333,281,137 (+)357	C_23_H_28_O_12_	Oxypaeoniflora
17	9.49	289.0736	289.0718	0.62				(−)245,203,123,	C_15_H_14_O_6_	Cianidanol
18	10.2	183.0312	183.0299	0.71				(−)124	C_8_H_8_O_5_	Methyl gallate
19	10.2	183.031	183.0299	0.6				(−) 140,124	C_7_H_6_O_3_	4-Hydroxybenzoic acid
20	11.02	563.1412	563.1406	0.11				(−)383,353	C_26_H_28_O_14_	Isoschaftoside
21	11.07	210.0785	210.0761	0				(−) 164,124,	C_9_H_11_NO_2_	Phenylalanine
22	11.73	525.1615	525.1603	0.23				(−)449,327,165,121	C_23_H_28_O_11_	Paeoniflorin
23	12.23	463.0896	463.0882	0.3				(−)300	C_21_H_20_O_12_	Hyperoside
24	12.29	525.307	525.3058	0.23				(−)479,319,159	C_27_H_44_O_7_	Hydroxyecdyone
25	12.29	447.09	447.0933	−0.74				(−) 356,285	C_21_H_20_O_11_	Astragalin
26	12.44	525.3071	525.3058	0.25				(−)479	C_27_H_44_O_7_	Rhapontisterone
27	12.77	197.0471	197.0444	1.4				(−)169,162,152,124	C_8_H_8_O_3_	Vanillin
28	13.04	807.2765	807.2717	0.59				(−)645,514,351	C_38_H_48_O_19_	Epimedin B
29	13.25	193.0517	193.0506	0.57	217.0467	217.0471	−0.18	(−)121(+)134	C_10_H_10_O_4_	Ferulic acid
30	13.3	525.1606	525.1603	0.06				(−)479,357,121	C_23_H_28_O_11_	Albiflorin
31	13.33	389.1267	389.1242	0.64				(−)227,185,143	C_20_H_22_O_8_	Polydatin
32	13.56	837.2856	837.2881	−0.31				(−)675	C_39_H_50_O_20_	Epimedin A
33	13.56	867.2963	867.2917	0.53				(−)679,367	C_39_H_50_O_19_	Epimedin C
34	13.58	675.2321	675.2294	0.4				(−)366,351	C_33_H_40_O_15_	Icariin
35	14.21	431.0988	431.0984	0.09	455.0946	455.0949	−0.07	(−)269,225 (+)293,185,164	C_21_H_20_O_10_	Apigenin-7-*O*-2te2glucopyranoside
36	14.23	431.1015	431.0984	0.72	455.0969	455.0973	−0.88	(−)269,225 (+)293,185	C_21_H_20_O_10_	Emodin-8-*O*-β-d-glucoside
37	14.26	955.4958	955.4908	0.52				(−)793	C_48_H_76_O_19_	Ginsenoside-Ro
38	14.31	285.0409	285.0405	0.14				(−)133	C_15_H_10_O_6_	Luteolin
39	14.35	301.0354	301.036	−0.2				(−)282,229,151	C_15_H_10_O_7_	Quercetin
40	14.66	629.1877	629.1865	0.19	607.1845	607.1786	0.97	(−)583, (+)607,341,289,105	C_30_H_32_O_12_	Benzoylpaeoniflorin
41	14.99	299.056	299.0561	0.03				(−)284	C_16_H_12_O_6_	Kaempferol
42	15.01	299.0558	299.055	0.27				(−)284	C_15_H_10_O_4_	Chrysophonal
43	15.01	299.0558	299.0561	−0.1				(−)284	C_16_H_12_O_6_	Fallacinol
44	15.07				215.0324	215.0315	0.42	(+)140	C_10_H_8_O_4_	Scopoletin
45	15.62				229.0861	229.0859	0.87	(+)211,165,152,135,119,107	C_14_H_12_O_3_	Resveratrol
46	16.04	513.1787	513.1766	0.41				(−)366,351,323	C_27_H_30_O_10_	IcarisideII
47	16.11				247.0656	247.0577	3.2	(+) 140,105	C_11_H_12_O_5_	Sinapic
48	16.14	205.0884	205.087	0.68				(−)161	C_12_H1_4_O_3_	Chuanxiongol
49	16.46	323.2234	323.2228	0.19				(−)305,265	C_18_H_30_O_2_	Linolenic acid
50	16.51				211.0648	211.0754	−5.01	(+)181,163,135,120, 105	C_12_H_12_O_2_	*n*-Butylidenephthalide
51	16.52	283.0624	283.0612	0.42				(−)240,212,183	C_16_H_12_O_5_	Physcion
52	16.55				247.0941	247.0946	−0.2	(+)229,113	C_12_H_16_O_4_	SenkyunolideI
53	16.61				217.0508	217.0495	0.6	(+)152,123	C_12_H_8_O_4_	Isobergapten
54	16.63				163.0411	163.039	1.3	(+)135,107,105	C_9_H_6_O_3_	Umbelliferone
55	16.66				269.0828	269.0784	1.6	(+)205,188	C_14_H_14_O_4_	Columbianetin
56	16.66	241.1441	241.1434	0.27				(−)225,197	C_12_H_20_O_2_	l-Bornyl acetate
57	16.79				187.0412	187.039	1.2	(+)131,115	C_11_H_6_O_3_	Psoralen
58	16.85	269.0831	269.0819	0.45				(−)254,225,210	C_16_H_14_O_4_	Imperatorin
59	16.85	251.1652	251.1642	0.41				(−)152,133	C_14_H_22_O	2,6-Di-*tert*-butylphenol
60	16.9	251.167	251.1641	1.12				(−)209,151	C_14_H_22_O	2,4-Di-*tert*-butylphenol
61	16.98	255.0674	255.0663	0.43				(−)201,166	C_15_H_12_O_4_	Isoliquiritigenin
62	17.03				284.0973	284.0989	−0.56	(+) 239,185	C_10_H_13_N_5_O_5_	Guanosine
63	17.04				187.0421	187.039	1.66	(+)118	C_11_H_6_O_3_	Isopsoralen
64	17.31	239.13	239.1278	0.92				(−)223,195,139	C_12_H_18_O_2_	Sedanolide
65	17.43				217.0509	217.0495	0.65	(−)202,174,145	C_12_H_8_O_4_	Bergapten
66	17.65	269.0823	269.0819	0.15				(−)241,225	C_16_H_14_O_4_	Isoimperatorin
67	17.66	269.0465	269.0455	0.37				(−)241,225	C_15_H_10_O_5_	Frangulic acid
68	17.75	269.0469	269.0455	0.52				(−)225	C_15_H_10_O_5_	Apigenin
69	17.79	315.2445	315.2541	−3				(−)297,279,171,141	C_17_H_34_O_2_	Heptadecanoic
70	17.8	315.2562	315.2529	1				(−)297,279	C_17_H_34_O_2_	Methyl palmitate
71	17.95				193.1224	193.1223	0.05	(+)175,147,137,105	C_12_H_16_O_2_	Senkyunolide A
72	18.17	301.2398	301.2373	0.82				(−)239,169	C_16_H_32_O_2_	Palmitic acid
73	18.2	277.1474	277.1445	1.5	301.1463	301.141	1.8	(−)147,134,121, (+)245	C_16_H_22_O_4_	Dibutyl phthalate
74	18.25				191.1062	191.1067	−0.26	(−)173,161,145,130,115,105	C_12_H_14_O_2_	Ligustilide
75	18.62				273.1124	273.1097	0.99	(+)241,140,105	C_14_H_18_O_4_	Dipropylphtalate
76	18.87				268.1068	268.104	1	(+)119,105	C_10_H_13_N_5_O_4_	Adenosine
77	18.88				245.121	245.1172	1.51	(+)189,131	C_15_H_16_O_3_	Osthole
78	18.91	353.2713	353.2686	0.75				(−)335,239,211,183	C_20_H_36_O_2_	Ethyl linoleate
79	18.91				189.0543	189.0522	1.11	(+)152,131,115,103	C_9_H_10_O_3_	Paeonol
80	19.05	199.1335	199.1329	0.3				(−)164	C_10_H_18_O	Eucalyptol
81	19.11				329.1372	329.1384	−0.36	(+)229,187,175,159,131	C_19_H_20_O_5_	Columbianadin
82	20.26				381.206	381.206	0	(+)231,189	C_24_H_28_O_4_	Levistilide A
83	20.28				335.1915	335.1853	1.8	(+)317,207	C_19_H_26_O_5_	Rubrosterone
84	20.69				577.4143	577.4463	−5.51	(+)560,448,278,234,133	C_35_H_60_O_6_	Daucosterol
85	20.85	455.3541	455.3531	0.22				(−)391	C_30_H_48_O_3_	Oleanolic acid
86	21.48				463.3024	463.3054	−0.65	(+)337,319	C_27_H_42_O_6_	Stachysterone D
87	22.59				353.2686	353.2662	0.68	(+)186	C_19_H_38_O_4_	Monopalmitin
88	24.66	281.2496	281.2486	0.36				(−)223,207	C_18_H_34_O_2_	Oleic acid

### Compounds selection for quantitative analysis

As shown in the literature, catechin could reduce inflammation and slow cartilage breakdown (Adcocks et al. 2002), paeoniflorin could down regulate the levels of TNF-α and myeloperoxidase, and reduce the production of IL-6 in LPS-simulated mouse macrophage RAW264.7 cells (Zhang et al. 2014). Other research indicated that paeoniflorin inhibited intercellular adhesion molecule-1 expression in LPS-treated U937 cells and TNF-α-stimulated human umbilical vein endothelial cells by suppressing the activation of the NF-κB pathway (Jin et al. 2011). Hyperoside could significantly decrease the mRNA expression and production of IL-1β, IL-6 in stimulated HMC-1 cells (Han et al. 2014). Ferulic acid may offer beneficial effects against osteoarthritis (Li et al. 2011), and ferulic acid has chondroprotective effects on hydrogen peroxide-stimulated chondrocytes through depressing hydrogen peroxide-induced pro-inflammatory cytokines and metalloproteinase gene expression at the mRNA level (Chen et al. 2010). Polydatin has efficacious anti-inflammatory activity by attenuating the phosphorylation of ERK1/2, JNK, and p38 (Lou et al. 2015). Quercetin could ameliorate all markers of inflammation (Gardi et al. 2015). Research suggested that MAPK signaling factors were involved in inflammation, quercetin inhibited the MAPK signal factors in macrophages, and quercetin also inhibited the secretion of the inflammatory cytokines IL-1β, IL-6, and stimulated the anti-inflammatory cytokine IL-10 (Seo et al. 2015). Based on the research, proinflammatory cytokines in the cartilage and synovium will stimulate their own production and induce chondrocytes to produce some abnormal biomechanical forces, such as proteases, chemokines, and nitric oxide, which will result in an imbalance between the chondrocyte anabolic and catabolic pathways, and ultimately leads to progressive joint destruction. Resveratrol keeps chondrocyte from apoptosis and reverses the catabolic state of chondrocytes in OA pathway (Dave et al. 2008). Due to its antiapoptotic, anti-inflammatory, and antioxidant properties, resveratrol have anti-osteoarthritic effects (Shen et al. 2012). Inflammatory cytokine IL-1β is one of the key inflammatory factors in intervertebral disc degeneration, psoralen could remit the degeneration of intervertebral disc chondrocyte induced by IL-1β (Yang et al. 2015), it also significantly suppressed T helper 2 cytokines of IL-4, IL-5, and IL-13 by ConA-stimulated D10 cells without inhibitory effect on cell viability (Jin et al. 2014). Isopsoralen was being used for its central inhibitory activities and inhibitory role in cell proliferation and antimicrobial (Liu et al. 2013). Icariside II exhibits anti-inflammatory activity, but its molecular pathways in human cells are poorly understood (Kim et al. 2011). Osthole can prevent isoprenalin-induced myocardial fibrosis in mice, and the mechanisms perhaps related to the reduction of TGF-β1 expression (Chen et al. 2011), osthole could also enhance osteoclasts apoptotic and inhibit the bone resorption through RANK + RANKL/TRAF6/Mkk/JNK signal pathway (Ming et al. 2012). TNF-α is a major inflammatory cytokine that mediates immune responses and systemic inflammation. Isoimperatorin inhibits TNF-α-induced expression of VCAM-1, therefore, isoimperatorin can be used for the treatment of pathologic inflammatory disorders (Moon et al. 2011).

**Figure 2.**
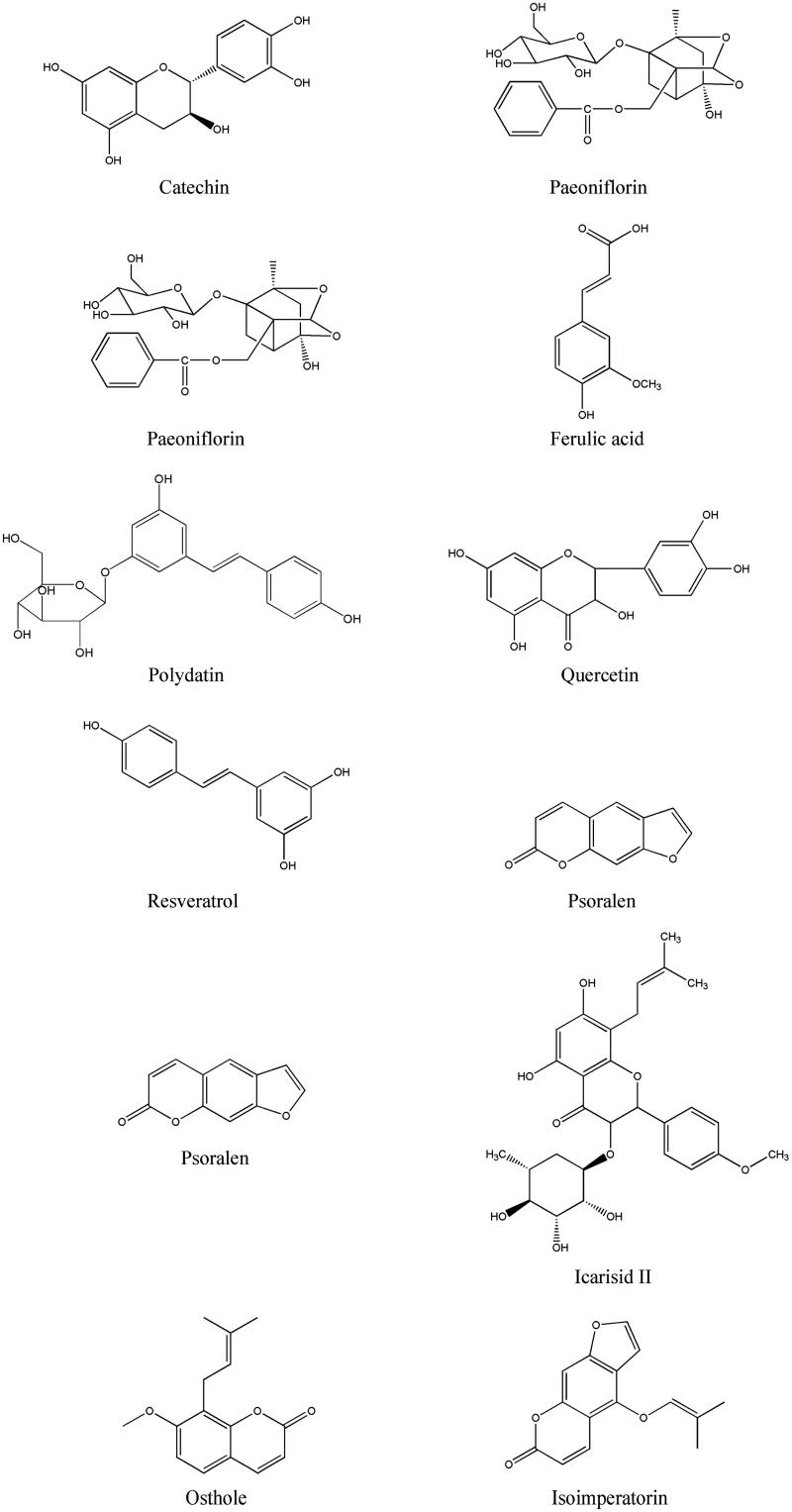
Chemical structures of 12 compounds determined simultaneously.

Through the review of the literature, we selected above described 12 active compounds with potential in treating OA and inhibiting chondrocyte apoptosis from these 88 compounds above, which were identified by HPLC-ESI-Q-TOF-MS for qualitative analysis. Chemical structures of these 12 compounds are shown in Figure 2.

### Quantitative analysis

**Figure 3.**
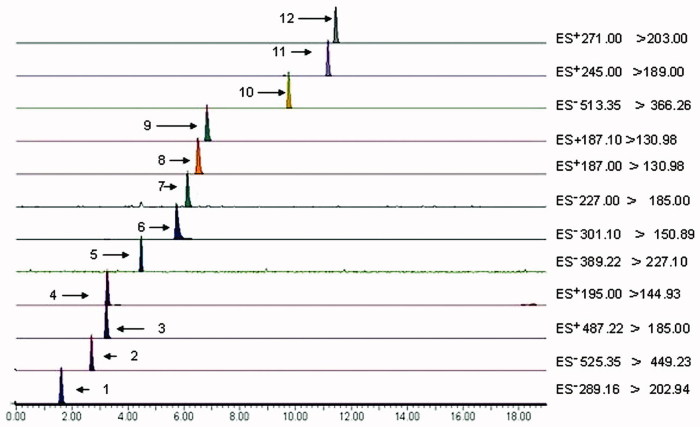
LC-MS/MS chromatogram of the 12 compounds in BSHXD. Retention time (RT): **1**→Catechin (1.61 min), **2**→Paeoniflorin (2.70 min), **3**→Hyperoside (3.23 min), **4**→Ferulic acid (3.28 min), **5**→Polydatin(4.43 min), **6**→Quercetin (5.73 min), **7**→Resveratrol (6.09 min), **8**→Psoralen (6.50 min), **9**→Isopsoralen (6.82 min), **10**→IcarisideII (9.75 min), **11**→Osthole (11.16 min), and **12**→Isoimperatorin (11.44 min).

UPLC-ESI-TQ-MS technology was used to determine the contents of catechin, paeoniflorin, hyperoside, ferulic acid, polydatin, quercetin, resveratrol, psoralen, isopsoralen, icariside II, osthole, and isoimperatorin. LC-MS/MS chromatogram of the 12 compounds in BSHXD is given in Figure 3, and their contents in the single herbs and decoction were summarized in Table 2. The standard curves and linear ranges of these 12 compounds are shown in Table 3. The precision and the accuracy were validated by the determination of the peak areas of compounds of interest during the preparation procedure. Relative standard deviation (RSD) of each compound was lower than 3%, the results showed that the method displays good precision and accuracy for each compound. The stabilities of these 12 compounds were tested at 0, 2, 4, 6, 12, and 24 h, RSD values of the peak areas were all no more than 3%, that revealed that the compounds were stable within 24 h. BSHXD sample was made into six solutions, inject 2 μL each time, and MS chromatography was used to determine the contents of each compound. The average content of catechin was 0.0411 mg/g, paeoniflorin was 4.2455 mg/g, hyperoside was 0.1199 mg/g, ferulic acid was 0.0576 mg/g, polydatin was 0.7589 mg/g, quercetin was 0.2001 mg/g, resveratrol was 0.1567 mg/g, psoralen was 0.8863 mg/g, isopsoralen was 0.4598 mg/g, icariside II was 0.0919 mg/g, osthole was 0.5274 mg/g, and isoimperatorin was 0.1490 mg/g, and RSD values were all less than 3%, indicating that the method was stable.

**Table 2.** Content determination of the samples (mg/g, *n* = 3).

Compounds	*Angelicae Pubescentis Radix*	*Taxilli Herba*	*Achyranthis Bidentatae Radix*	*Epimedii Folium*	*Chuanxiong Rhizoma*	*Angelicae Sinensis Radix*	*Paeoniae Radix Alba*	*Polygoni Cuspidati Rhizoma Et Radix*	*Arisaematis RhizomaPreparatum*	BSHXD
Catechin								0.0489		0.0422
Paeoniflorin							5.2222			4.2596
Hyperoside				0.7591						0.1211
Ferulic acid					0.0411	0.0913				0.0584
Polydatin								12.5807		0.7594
Quercetin		0.0662						0.0101		0.1999
Resveratrol								0.5818		0.1570
Psoralen	0.9954									0.8948
Isopsoralen	0.3765									0.4634
IcarsideII				0.0322						0.0928
Osthole	0.1087									0.5364
Isoimperatorin	0.0763									0.1487

**Table 3.** Standard curve and linear range of each compound.

Number	Rt (min)	Compounds	Regression equation	*R*^2^	Linear range (μg/mL)
1	1.61	Catechin	*Y* = 6079.2*x* − 204.2	0.9998	0.0106–3.5698
2	2.70	Paeoniflorin	*Y* = 5320.8*x* + 114.38	0.9997	0.0404–14.074
3	3.23	Hyperoside	*Y* = 6692.6*x* − 595.73	0.9999	0.0994–3.8286
4	3.28	Ferulic acid	*Y* = 19721*x* − 1927.6	0.9984	0.0987–1.8724
5	4.43	Polydatin	*Y* = 16912*x* − 226.29	0.9990	0.0100–4.5306
6	5.73	Quercetin	*Y* = 50207*x* − 1707	0.9993	0.0350–3.9307
7	6.09	Resveratrol	*Y* = 1689.5*x* − 70.719	0.9998	0.1259–3.8672
8	6.50	Psoralen	*Y* = 273637*x* − 3207.9	0.9978	0.0196–4.7626
9	6.82	Isopsoralen	*Y* = 336719*x* − 9923.6	0.9999	0.0400–3.8719
10	9.75	Icariside II	*Y* = 271366*x* − 3081.3	0.9996	0.0100–0.9741
11	11.16	Osthole	*Y* = 1233900*x* − 69509	0.9989	0.0201–5.2790
12	11.44	Isoimperatorin	*Y* = 88731*x* − 7657.9	0.9979	0.0708–1.9528

### ELISA results

TNF-α, IL-6, and NO play an important role in cells, tissues, and organs, respectively. Within cartilage, pro-inflammatory cytokines such as TNF-α auto-catalytically stimulate its production and induce chondrocytes to produce additional catabolic mediators that abnormal biomechanical forces will lead to progressive joint destruction (Abramson & Yazici 2006). IL-6-signal transducer may conduce to the posttraumatic development of osteoarthritis (Liu et al. 2015), and IL-6 can be considered as a marker of nerve injury and proinflammatory cytokines which produced by joint tissue (Malek et al. 2015). Imbalance of catabolic and anabolica factors including cytokines and NO could result in OA (Chevalier et al. 2013). In that case, TNF-α, IL-6, and NO were utilized to explore the mechanism of BSHXD in treating OA. Changes of released inflammatory mediators’ concentration were observed by ELISA. It is shown that quercetin, isopsoralen, icariside II, osthole, isoimperatorin, and BSHXD have different effects in inhibiting the release of TNF-α, IL-6, and NO. Quercetin (100 μg/mL), isopsoralen (100 μg/mL), icariside II (100 μg/mL), osthole (20 and 100 μg/mL), isoimperatorin (100 μg/mL), and BSHXD (100 μg/mL) had a significant inhibition effect on the release of TNF-α, *p* < 0.01; quercetin (20 and 100 μg/mL), isopsoralen (100 μg/mL), icariside II (100 μg/mL), osthole (20 and 100 μg/mL), and isoimperatorin (20 and 100 μg/mL) had a significant inhibition effect on the release of IL-6, *p* < 0.01; five compounds (20 and 100 μg/mL) and BSHXD (100 μg/mL) had a remarkable inhibition effect on the release of NO, *p* < 0.01. The results showed that the monomers hold generally stronger inhibition effect than BSHXD. Tables 4–6 describe the detailed inhibition results of these components of decoction on TNF-α, IL-6, and NO released by RAW264.7 cell after induction of LPS.

**Table 4.** The inhibition of different components on TNF-α released by RAW264.7 cell after induction of LPS.

Group	Concentration (μg/mL)	Inhibition (%)
Control	–	–
LPS	0.5	–
Quercetin	20	5.86
	100	57.68**
Isopsoralen	20	16.48*
	100	64.38**
IcarisidII	20	58.17**
	100	103.79**
Osthole	20	90.58**
	100	104.05**
Isoimperatorin	20	17.54*
	100	49.4**
BSHXD	20	1.54
	100	35.75**

x ± s, *n* = 3, groups compared with the LPS model control group.

* *p* < 0.05.

** *p* < 0.01.

**Table 5.** The inhibition of different components on IL-6 released by RAW264.7 cell after induction by LPS.

Group	Concentration (μg/mL)	Inhibition (%)
Control	–	–
LPS	0.5	–
Quercetin	20	29.45**
	100	79.73**
Isopsoralen	20	18.66*
	100	91.15**
IcarisidII	20	1.64
	100	99.03**
Osthole	20	99.37**
	100	100.03**
Isoimperatorin	20	64.23**
	100	67.39**
BSHXD	20	0.60
	100	13.11*

x ± s, *n* = 3, groups compared with the LPS model control group.

* *p* < 0.05.

** *p* < 0.01.

**Table 6.** The inhibition of different components on NO released by RAW264.7 cell after induction by LPS.

Group	Concentration (μg/mL)	Inhibition (%)
Control	–	–
LPS	0.5	–
Quercetin	20	73.18**
	100	77.29**
Isopsoralen	20	93.46**
	100	81.31**
IcarisidII	20	61.03**
	100	89.44**
Osthole	20	77.29**
	100	89.44**
Isoimperation	20	52.90**
	100	65.05**
BSHXD	20	16.26*
	100	56.92**

x ± s, *n* = 3, groups compared with the LPS model control group.

* *p* < 0.05.

** *p* < 0.01.

## Conclusion

According to LC-MS/MS analysis, 88 compounds from BSHXD were confirmed. Twelve compounds which have a potential role in treating OA were selected and quantified. By comparing the contents of 12 compounds in BSHXD and single herbs, we found that five of them increased significantly. Therefore, the anti-inflammatory activity *in vitro* was tested. ELISA was used to detect the effect of quercetin, isopsoralen, icariside II, osthole, isoimperatorin, and BSHXD on TNF-α, IL-6, and NO released by macrophage, we found that the compounds had some or remarkably inhibitory effect on the former cytokines, which may demonstrate the possible reason and mechanism of BSHXD in treating OA.

In traditional Chinese medicine theory, the fact that different herbs used in combination can enhance the therapeutic efficacy compared with those when they were used separately is called ‘Xiang Xu’. In BSHXD, nine herbs which have different therapeutic effects were decocted together, and the results of UPLC-ESI-TQ-MS showed the contents of compounds of interest increased or decreased, which may be due to certain chemical reactions occurred among the chemical constituents in the herbs. That means that the contents of some compounds which have potential therapeutic effects on OA were higher in decoction than in single herb that may give rise to reinforcement of therapeutic effects on OA.
